# Time‐effectiveness and convenience of transvaginal ultrasound probe disinfection using ultraviolet *vs* chlorine dioxide multistep wipe system: prospective survey study

**DOI:** 10.1002/uog.24834

**Published:** 2022-07-01

**Authors:** C. Kyriacou, E. Robinson, J. Barcroft, N. Parker, M. Tuomey, C. Stalder, D. Gould, M. Al‐Memar, T. Bourne

**Affiliations:** ^1^ Tommy's National Centre for Miscarriage Research, Department of Obstetrics and Gynaecology Queen Charlotte's & Chelsea Hospital, Imperial College London London UK; ^2^ St Mary's Hospital, Department of Obstetrics and Gynaecology Imperial College London London UK; ^3^ Department of Obstetrics and Gynecology University Hospitals Leuven Leuven Belgium

**Keywords:** chlorine dioxide, gynecology, health and safety, ultrasonography, ultraviolet

## Abstract

**Objectives:**

To compare the efficiency, ease of use and user satisfaction of two methods of transvaginal ultrasound probe high‐level disinfection: ultraviolet‐C radiation (UV‐C) and a chlorine dioxide multistep wipe system.

**Methods:**

This was a prospective survey study. UV‐C units were introduced into a busy early pregnancy assessment service and compared with a multiwipe system for disinfection. Before seeing each patient, healthcare professionals (HCPs) measured with a stopwatch the time taken to complete a cycle of disinfection using either UV‐C or chlorine dioxide multistep wipes and responded to a quick‐response (QR) code‐linked survey. Additional essential tasks that could be completed before seeing the next patient during probe disinfection were also documented. Using another QR code‐linked survey, data on ease of use, satisfaction with the system used and preferred system were collected. The ease of use and satisfaction with the system were rated on a 0 to 10 Likert scale (0 poor, 10 excellent). A free‐text section for comments was then completed.

**Results:**

Disinfection using UV‐C (*n* = 331) was 60% faster than the chlorine dioxide multiwipe system (*n* = 332) (101 *vs* 250 s; *P* < 0.0001). A greater number of tasks were completed during probe disinfection when using UV‐C, saving a further 74 s per patient (*P* < 0.0001). The HCPs using UV‐C (*n* = 71) reported greater ease of use (median Likert score, 10 *vs* 3; *P* < 0.0001) and satisfaction (median Likert score, 10 *vs* 2; *P* < 0.0001) compared with those using the multiwipe system (*n* = 43). HCPs reported that the chlorine dioxide system was time‐consuming and environmentally unfriendly, while the UV‐C system was efficient and easy to use. Overall, 98% of the HCPs preferred using the UV‐C system.

**Conclusions:**

UV‐C technology is more time‐efficient and allows more essential tasks to be completed during disinfection. For a 4‐h ultrasound list of 15 patients, the use of UV‐C would save 55 min 45 s. HCPs found UV‐C preferable and easier to use. © 2021 The Authors. *Ultrasound in Obstetrics & Gynecology* published by John Wiley & Sons Ltd on behalf of International Society of Ultrasound in Obstetrics and Gynecology.

## Abstract 2

Linked article: There is a comment on this article by Swinney et al. Click here to view the Correspondence.

**CONTRIBUTION**

**What are the novel findings of this work?**

Compared with the chlorine dioxide multistep wipe system, ultraviolet technology is a more time‐efficient method of transvaginal ultrasound probe disinfection and allows more essential tasks to be completed during probe disinfection. For a 4‐h ultrasound list of 15 patients, 55 min 45 s extra time can be saved using the ultraviolet disinfection approach. Healthcare professionals preferred the ultraviolet technology and found it easier to use than the chlorine dioxide multistep wipe system.

**What are the clinical implications of this work?**

The additional time saved when utilizing ultraviolet technology may allow approximately three additional patients to be seen per ultrasound list, amounting to six extra patients per day. This would potentially increase patient throughput, reduce waiting times and, in some healthcare settings, increase the income a unit can generate.

## INTRODUCTION

Transvaginal ultrasound (TVS) probes, used repeatedly in early pregnancy and gynecology clinical sessions, are considered to be semicritical devices owing to their contact with mucous membranes^1^. To provide safe care, consistent and thorough high‐level disinfection (HLD) of such devices is necessary owing to the persistent survival of infective bacteria and viruses on probes, probe covers and ultrasound gel following use^1^, ^2^, ^3^, ^4^, ^5^, ^6^, ^7^.

Contamination following disinfection is likely if barrier protection and transducer hygiene practices are poor^8^. Multiple pathogens have been identified on probes following disinfection, including methicillin‐resistant *Staphylococcus aureus* and human papillomavirus (HPV)^6^, ^9^, ^10^, ^11^. The concerning HPV strains, types 16 and 18, are the most common causes of cervical cancer and are highly resistant to decontamination^4^, ^10^, ^11^.

Multiple probe HLD techniques exist. Manual reprocessing methods utilize baths of chemicals for disinfection, such as glutaraldehyde or ortho‐phthalaldehyde^3^, ^12^, ^13^, ^14^. These methods necessitate 10–20 min per disinfection, require chemical neutralization before disposal, carry a risk of operator injury through skin, mucous membrane or pulmonary irritation and are ineffective at eradicating HPV types 16 and 18^3^, ^12^, ^15^.

Disposable single or multistep wipes utilize chemicals such as chlorine dioxide. They are reported to require 2–3 min per disinfection and are effective at eradicating pathogens, including HPV^16^, ^17^, ^18^, ^19^, ^20^, ^21^. Although chlorine dioxide is toxic, single and multistep wipes are easier to dispose of and are safer than chemical baths^16^, ^17^, ^18^, ^19^, ^22^.

The lack of training or time to use correctly the abovementioned methods may result in inadequate HLD^1^, ^4^, ^23^, ^24^, ^25^. Automated hydrogen peroxide devices used within a ‘sonically activated’ closed system (instead of manual reprocessing) reduce chemical exposure and have high antimicrobial effectiveness but take 7 min per cycle^3^, ^12^, ^26^, ^27^, ^28^. Automated ultraviolet‐C radiation (UV‐C) devices offer safe and reusable chemical‐free HLD, are highly effective at eradicating pathogens, including HPV, and take 90 s per cycle^1^, ^4^, ^24^, ^29^, ^30^, ^31^, ^32^, ^33^.

We aimed to compare the time‐effectiveness and convenience of UV‐C and a chlorine dioxide multistep wipe system, the two methods used at our center for TVS‐probe HLD.

## METHODS

### Study design and setting

This report has been written in accordance with the STROBE cross‐sectional study statement^34^. This was a prospective survey study comparing a UV‐C system (Hypernova Chronos; Germitec, Ivry‐sur‐Seine, France) and a chlorine dioxide multistep wipe system (Tristel Trio; Snailwell, UK) for TVS‐probe HLD. Both systems were used clinically by healthcare professionals (HCPs) in our busy central London early pregnancy assessment service.

The UV‐C devices are chemical‐free and were positioned adjacent to the TVS machine. After removing the probe cover, the probes were wiped using a dry tissue to remove any excess gel from the probe. There was a low threshold to use a hospital‐ and manufacturer‐approved disinfection wipe (Clinell wipes; GAMA Healthcare Ltd, Hemel Hempstead, UK) to ensure that the probe was visibly clean. The HCP then disposed of their gloves, cleaned their hands, put on replacement gloves and carefully inserted the probe, handle and distal aspect of the cord into the module of the UV‐C device. As gloves were disposed of and hands cleaned, the machine scanned the probe's unique barcode. Once the door was completely shut and the barcode scanned (to confirm that the probe had been placed correctly and for traceability purposes), the HCP pressed a button on the front of the device to trigger consistent release of UV‐C for a predefined period. The front of the device flashed blue during this time. Once the flashing stopped, a sticker was printed automatically by the device, confirming completion of the disinfection cycle.

The chlorine dioxide approach is a chemical‐based three‐wipe system. After removing the probe cover, the probes were wiped using a dry tissue to remove any excess gel from the probe. There was a low threshold to use a hospital‐ and manufacturer‐approved disinfection wipe (Clinell) to ensure that the probe was visibly clean. The first wipe was then used once the examiner's gloves had been disposed of, hands cleaned and gloves replaced. The first wipe contained a triple enzymatic detergent and surfactant, used to preclean the probe. Gloves were again disposed of, hands cleaned and gloves replaced before using the second wipe. This had a sporicidal HLD component, which generated chlorine dioxide when combined with a custom activator foam for the requisite 15 s. The foam remained on the probe for 30 s. Although not compulsory, during this time, some HCPs cleaned their hands and replaced gloves before using the third wipe. The third wipe was impregnated with deionized water to rinse and remove chemical residue. Disinfection traceability was performed manually using a record book once gloves had been disposed of and hands cleaned for the final time.

The HCPs were trained in the use of both systems before being allocated a room for each scanning session. One of the two HLD systems was placed in each room. Two quick‐response (QR) code‐linked surveys were made available for the purposes of data collection using Qualtrics^XM^ (2021) (Provo, UT, USA). The first survey was performed before seeing each patient and the second survey was performed after each TVS session. We estimated that the time taken to complete each survey was 30 s.

### Participants

HCPs trained in TVS participated in the study from November 2020 to March 2021. Before seeing each patient, the HCP would scan the first QR code on their phone and document timings recorded on a stopwatch in addition to answer a multiple‐choice questionnaire (the ‘scan’ survey). At the end of each session, the HCP would scan a second QR code and answer the questions (the ‘session’ survey). Participation was voluntary and responses were anonymous. Bias was avoided by the HCPs submitting responses to the questions using their personal hand‐held device following each HLD and scanning session while still in their scanning room.

The power calculation for the scan survey hypothesized that the UV‐C system would be 30% more efficient than the chlorine dioxide wipes. For the study to be adequately powered and for a *P*‐value of 0.05 to be deemed significant, a minimum of 175 responses were required for each system. The number of session surveys was defined by the number of scan surveys collected.

### Variables and data sources

The scan survey was performed before seeing each patient and recorded the time taken to complete a cycle of disinfection, as well as additional necessary tasks that could be completed before seeing the next patient during probe disinfection (Appendix S1). The tasks essential for completing a patient appointment (other than HLD) included: (1) cleaning the bed; (2) finishing the administrative paperwork (e.g. the TVS report); (3) loading the next patient's details and worklisting them for TVS; (4) reviewing their notes; (5) loading their demographic details onto the ultrasound machine; and (6) calling them to the scanning room. In a separate analysis, these tasks were timed seven times by one of two HCPs proficient in TVS to calculate the average time each activity should take. This allowed us to approximate how much time was saved when one or more of these jobs were completed during probe disinfection, as it was not feasible for the HCP to time each of these additional tasks at the same time as timing HLD during their scanning sessions.

The session survey was completed at the end of each TVS session. The HCP scored ease of use and satisfaction with the system used on a 0 to 10 Likert scale (0 poor, 10 excellent). They then selected their preferred HLD system before being offered a free‐text section in which subjective comments could be added (Appendix S2).

### Ethical approval

As this was a survey study involving HCPs at a local unit, comparing the current trust standard of chlorine dioxide wipes, ethical approval was not required. The study was registered as an audit at Imperial College Healthcare National Health Service (NHS) Trust (audit number: GRM_073).

### Outcome measures

The main differentiator throughout was the HLD system used. The primary outcome measure was the time taken for HLD to be performed. Secondary outcome measures included the number of tasks completed during HLD (and thus additional time saved), ease of use, satisfaction and preferred approach of the HCPs.

### Statistical analysis

Univariate analysis was performed comparing the time taken to complete HLD, the time saved per patient by completing other activities during probe HLD, ease of use, satisfaction and preference. Distribution was assessed using the D'Agostino–Pearson test.

Data were presented as median (interquartile range (IQR)) and analyzed using a two‐tailed Mann–Whitney *U*‐test. Statistical analysis was performed using GraphPad Prism version 8.2.1 (GraphPad Software, San Diego, CA, USA).

For the free‐text answers, a summary of the common themes is discussed without a formal qualitative analysis. This was owing to the limited number of comments.

## RESULTS

A total of 663 scan surveys were performed, including 332 (50.1%) for multistep chlorine dioxide wipes and 331 (49.9%) for UV‐C. There were 114 session surveys, including 43 (37.7%) following the use of chlorine dioxide wipes and 71 (62.3%) following the use of UV‐C (Tables 1 and 2). All surveys were eligible for analysis.

**Table 1:** Number of surveys completed (*n* = 663) and timings and additional tasks completed per high‐level disinfection (HLD) between patients for two HLD systems

Parameter	Chlorine dioxide wipes	UV‐C	*P*
Surveys completed	332 (50.1)	331 (49.9)	
Time to complete HLD (s)	250 (226–277)	101 (84–119)	< 0.0001
Additional tasks performed during HLD	118	412	< 0.0001
Clean patient bed	96/118 (81.4)	221/412 (53.6)	
Administrative work	8/118 (6.8)	115/412 (27.9)
Load next patient's details and worklist them	6/118 (5.1)	23/412 (5.6)
Review notes	1/118 (0.8)	43/412 (10.4)
Load next patient's details onto US machine	4/118 (3.4)	8/412 (1.9)
Call next patient	3/118 (2.5)	2/412 (0.5)
Mean time saved completing additional tasks per HLD (s)	29	152	
Time saved completing additional tasks during HLD (s)	0 (0–0)	74 (74–279)	< 0.0001
Time required to complete tasks in addition to HLD (s)	438 (438–438)	364 (159–364)	< 0.0001

Data are given as *n* (%), median (interquartile range), *n* or *n*/*N* (%), unless stated otherwise.

US, ultrasound; UV‐C, ultraviolet‐C radiation.

**Table 2:** Number of surveys completed per scanning session (*n* = 114) and ease of use, satisfaction and preference for two high‐level disinfection systems

Parameter	Chlorine dioxide wipes	UV‐C	*P*
Session surveys completed	43 (37.7)	71 (62.3)	—
Ease of use*	3 (2–4)	10 (10–10)	< 0.0001
Satisfaction*	2 (1–3)	10 (10–10)	< 0.0001
Preferred system	2 (1.8)	112 (98.2)	—

Data are given as *n* (%) or median (interquartile range).

* Ease of use and satisfaction with the system were rated using 0–10 Likert scale.

UV‐C, ultraviolet‐C radiation.

### Survey responses per scan (*n* = 663)

It took a median time of 250 s (IQR, 226–277 s) to complete multiwipe chlorine dioxide HLD compared with a median of 101 s (IQR, 84–119 s) necessary to complete UV‐C HLD (*P* < 0.0001) (Table 1). The use of UV‐C saved 149 s (2 min 29 s) per disinfection.

The average time taken to complete each task essential for every patient appointment (other than HLD) was: (1) 74 s to clean the bed; (2) 279 s to finish administrative paperwork; (3) 13 s to load the next patient's details and worklist them for TVS; (4) 37 s to review their notes; (5) 11 s to load their demographic details onto the ultrasound machine; and (6) 23 s to call them to the scanning room (Table 3).

**Table 3:** Average time taken to complete essential activities before seeing next patient, in addition to high‐level disinfection (HLD), according to one of two healthcare professionals (HCPs) performing seven scans

Additional task performed during HLD	Measurements (*n*)	Mean time taken to perform each task (s)
Clean patient bed	7	74.4
Administrative work	7	279.4
Load next patient's details and worklist them	7	13.3
Review notes	7	36.9
Load next patient's details onto US machine	7	10.7
Call next patient	7	23.3

US, ultrasound.

The HCPs managed to perform a total of 118 additional tasks during multiwipe chlorine dioxide HLD: (1) 96 cleaned the bed; (2) eight completed administrative paperwork; (3) six loaded the next patient's details and worklisted them for TVS; (4) one reviewed their notes; (5) four loaded their demographic details onto the ultrasound machine; and (6) three called them to the scanning room (Table 1). The total time saved by performing additional tasks during HLD amounted to 9564 s (159 min 24 s), with an average of 29 s saved during each of the 332 HLD sessions.

During UV‐C HLD, the HCPs performed 412 additional tasks: (1) 221 cleaned the bed; (2) 115 completed administrative paperwork; (3) 23 loaded the next patient's details and worklisted them for TVS; (4) 43 reviewed their notes; (5) eight loaded their demographic details onto the ultrasound machine; and (6) two called them to the scanning room (Table 1). The total time saved by performing additional tasks during HLD amounted to 50 463 s (841 min 3 s), with an average of 152 s saved during each of the 331 HLD sessions.

In many cases, the HCP was able to complete more than one additional task during HLD. These combinations were thus compared between the two disinfection systems to provide an accurate comparison of timings. In most instances, no additional task was completed during chlorine dioxide HLD. However, at least one additional task was completed during most UV‐C HLD sessions. This allowed a median saving of 74 s (IQR, 74–279 s) per patient appointment (*P* < 0.0001), in addition to the 149 s saved during HLD using UV‐C (Table 1). As a result, the median time required to complete tasks between patients was 438 s (IQR, 438–438 s) using the multiwipe chlorine dioxide system compared with 364 s (IQR, 159–364 s) using UV‐C (*P* < 0.0001), as more tasks had already been completed during HLD (Table 1).

The median time to complete HLD and additional tasks per patient was 688 s (250 + 438 s) (11 min 28 s) using chlorine dioxide multiwipes compared with 465 s (101 + 364 s) (7 min 45 s) using UV‐C. The use of UV‐C saved a total of 223 s (3 min 43 s) per patient.

### Survey responses per session (*n* = 114)

The median Likert score for ease of use of the technique by the HCPs was 3 (IQR, 2–4) for chlorine dioxide multistep wipes compared with 10 (IQR, 10–10) for the UV‐C system (*P* < 0.0001) (Table 2).

User satisfaction was similarly low when using the multiwipe system, with a median Likert score of 2 (IQR, 1–3) compared with 10 (IQR, 10–10) when using UV‐C (*P* < 0.0001) (Table 2). Of the 114 responses, 112 (98.2%) preferred the UV‐C system.

The free‐text responses from the HCPs using either device were collated (Appendix S3). The main themes revolved around the multistep chlorine dioxide wipe system being time‐consuming and environmentally unfriendly. The HCPs were also concerned about chemical use and the subjectivity of the disinfection regimen. However, they complimented the efficiency, automation, reproducibility, simplicity and ease of use of UV‐C.

## DISCUSSION

We found that the UV‐C technology was preferred by the majority of HCPs, reporting its use to be easier and more satisfactory compared with the use of the chlorine dioxide multiwipe system. The UV‐C system was more efficient, saving 2 min 29 s of disinfection time per scan compared with the chlorine dioxide multiwipe system. The UV‐C approach saved a further 1 min 14 s per patient when considering the completion of other essential tasks during HLD. Thus, a total of 3 min 43 s can be saved per patient when UV‐C is used.

The main strengths of our study are the large number of results collected, particularly per scan, the relatively simple design, reducing the introduction of error, and the use of QR codes to record real‐time responses in a paperless fashion. As the difference in efficiency reported by our findings surpassed sample calculation predictions, the study is also adequately powered. Using Likert scales for ease of use and satisfaction enabled subjective opinions to be quantified in an objective and statistically comparable manner. The prospective nature of the work reduced potential bias by capturing HCPs' opinions at the time of HLD.

The weaknesses of the study relate to the low number of session responses, the variation in HLD timings and the difficulty in timing the additional tasks at the same time as HLD. The number of session surveys was expected to be lower than the number of responses per scan given the number of scans performed per session. For HLD timing, although users were appropriately trained, our data reflect the pressures of clinical practice probably leading to the variation seen for when timing could safely be commenced. As the HCPs could not feasibly time tasks in addition to HLD, these were calculated in a clinic by HCPs proficient in TVS to provide estimations of additional time that could be saved when utilizing UV‐C HLD.

### Time saved, cost‐effectiveness and automation

Clinicians in emergency departments and those in obstetrics and gynecology internationally have reported difficulties in adhering to current disinfection protocols, which take several minutes, delaying patient care and interrupting workflow^25^, ^27^. Although it has been estimated that the multiwipe system takes 2–3 min^20^, in clinical practice we have found this to be longer, especially when compared with the use of the UV‐C device. A typical ultrasound scan session within our unit comprises 15 patients over 4 h. This amounts to 37 min 15 s extra time available when using UV‐C over chlorine dioxide multiwipes for HLD. An additional 18 min 30 s were made available by completing other essential tasks during UV‐C HLD. The total time saved for such a list thus equates to 55 min 45 s. With this much time available, approximately three additional patients could be seen, amounting to six extra patients per day, potentially increasing patient throughput, reducing waiting times and, in some healthcare settings, increasing the income generated by a unit.

Hospitals may have varying cleaning recommendations prior to UV‐C disinfection. There was a low threshold to use a cleaning wipe in addition to dry tissue for probe cleaning prior to UV‐C in this study. As such, there are no hidden additional time costs necessary when using UV‐C HLD.

The benefits extend beyond early pregnancy. When considering the current issues with gynecology and gynecological oncology diagnostics in some countries and the need to increase capacity for urgent patient referrals (scheduled or unscheduled), the potentially increased clinic capacity associated with the use of UV‐C technology is clinically significant for patients^23^. However, although single‐use wipe systems are less cumbersome and may provide similar time benefits to those of UV‐C, effectiveness of both single‐ and multiwipe systems may be hindered by issues related to reproducibility and subjectivity. The resulting inconsistent and unreliable HLD may slow down workflow and compromise patient safety^1^, ^4^, ^20^, ^25^.

Cost evaluation of manual and multistep wipe HLD systems concluded that, while chemical baths may be cheaper, manual reprocessing is not as convenient as using chlorine dioxide wipes^19^. However, UV‐C automation was not considered.

Although an automated system, such as the UV‐C device used in this study, may have greater upfront costs given the initial investment in the device, the low maintenance costs provide greater cost‐effectiveness over time. The ability to see more patients adds further to the cost‐effectiveness of using automated UV‐C devices for HLD, while also allowing the provision of traceable, efficient, reproducible, objective and reliable HLD. Although this improves patient safety by helping to prevent user errors described above when using wipes, it is important to remember that pre‐disinfection wiping of the probe is essential to ensure that UV‐C is effective. This step can therefore also be prone to user error^3^, ^12^, ^24^, ^29^, ^30^, ^33^.

### Chemical exposure

HCP assessment performed during HLD found that chlorine dioxide exposure was below the local occupational limits when the wipe system was utilized^35^. However, chlorine dioxide solutions are known to be toxic and irritant, can cause severe skin burns and eye injury and come with warnings to use eye protection^22^. While the wipes are less odorous and more user‐friendly than ortho‐phthalaldehyde, UV‐C is chemical‐free, odorless and non‐corrosive^18^, ^20^, ^36^.

### Residual contamination and decontamination

Chlorine dioxide has well‐documented bactericidal, mycobacterial and sporicidal properties and has become a popular HLD method^20^, ^37^. Although studies have reported complete decontamination following the use of chlorine dioxide wipes, recent work suggests the ongoing presence of bacterial growth on some of the probes evaluated^18^, ^38^, ^39^. This may be due to poor reproducibility among HCPs using the chlorine dioxide wipes or the fact that the probe is returned to an environment that carries a risk of recontamination. Automated devices that protect the probe until it is ready for use and utilize UV‐C in a reproducible manner provide a feasible disinfection alternative that has been shown to be effective against resistant and pathogenic bacteria and viruses, including HPV, so long as there is strict adherence to the entire disinfection protocol (including the pre‐clean probe wiping)^10^, ^24^, ^30^, ^31^.

Our data show that the use of UV‐C HLD is more time‐efficient than chlorine dioxide multiwipes. UV‐C HLD becomes cost‐effective over time, providing a chemical‐free, user‐friendly and reliable alternative to TVS‐probe decontamination that is automated for both disinfection and traceability subject to strict disinfection protocol adherence. Although we did not focus on the microbial aspects of disinfection in this study, from a time‐effectiveness and convenience perspective, we conclude that UV‐C HLD is the preferred disinfection approach for TVS within our unit.

## ACKNOWLEDGMENTS

We acknowledge all the staff working in early pregnancy and gynecology at Imperial College Healthcare NHS Trust. C.K. is supported by Imperial Health Charity, grant number RFPrD1920/116. T.B. is supported by the NIHR Imperial Biomedical Research Centre, grant number IS‐BRC‐1215‐20013.

### Disclosure

Germitec provided the early pregnancy assessment service with two Germitec Chronos devices for trial in the clinical setting. The views expressed are those of the author(s) and not necessarily those of the National Health Service, the National Institute of Health Research or the Department of Health.

## Supporting information


**Appendix S1** Survey performed before each patient: ‘scan’ survey
**Appendix S2** Survey performed after scanning session: ‘session’ survey
**Appendix S3** Free‐text comments from healthcare professionals after their scanning sessions

## Data Availability

Research data are not shared.
