# Supplementary material for: Time‐effectiveness and convenience of transvaginal ultrasound probe disinfection using ultraviolet vs chlorine dioxide multistep wipe system: prospective survey study
Source: Ultrasound Obstet Gynecol. 2022 Jul 1;60(1):132–8. doi: 10.1002/uog.24834 (PMC9414347; doi:10.1002/uog.24834)
Supplement: Supplementary file 1 — Appendix S1 Survey performed before each patient: ‘scan’ survey Appendix S2 Survey performed after scanning session: ‘session’ survey Appendix S3 Free‐text comments from healthcare professionals after their scanning sessions [file UOG-60-132-s001.docx]

**Appendix S1**  Survey performed before each patient: ‘scan’ survey

**Q1: Confirm you are scanning in a room where Tristel wipes are/the Germitec device is used to clean the transvaginal ultrasound probe**

Yes

No

**Q2: Please select your role**

Nurse

SHO

SPR

CRF

Consultant

**Q3: Using the stopwatch, how long did it take for the probe to be cleaned:**

**START TIME = cleaning the gel off the probe with dry paper towels following use with last patient**

**END TIME = completion of recording the probe clean in the Tristel book/machine finishes clean**

**Please enter number of minutes and seconds as e.g. 1.50 = 1 minute 50 seconds.**

**Q4: In the time it took for the probe to be cleaned, what did you manage to achieve (please tick all that apply):**

Cleaning the bed

Completing previous Astraia report

Review next patients’ clinical questionnaire or past clinical entry

Loading next patient onto computer and work listing them

Finding next patient on USS machine

Calling next patient

None of the above

**Appendix S2** Survey performed after each scanning session: ‘session’ survey

**THIS SURVEY SHOULD ONLY BE TAKEN ONCE PER SESSION**

**Q1: Confirm you are scanning in a room where Tristel wipes are/the Germitec device is used to clean the transvaginal ultrasound probe**

Yes

No

**Q2: Please select your role:**

Nurse

SHO

SPR

CRF

Consultant

**Q3: On a scale of 1-10, please document the ease of using the Tristel Trio wipes/Germitec device (1 = unable to use; 10 = very easy)**

1 2 3 4 5 6 7 8 9 10

**Q4: On a scale of 1-10, please document your satisfaction with the Tristel Trio wipes/ Germitec device (1 = unable to use; 10 = very easy)**

1 2 3 4 5 6 7 8 9 10

**Q5: Do you prefer using the Germitec UV device or the Tristel Trio wipes?**

Germitec

Tristel Trio

**Q6: Any comments (positive or negative) regarding the Germitec device or Tristel Trio wipes? (not compulsory to complete):**

**Appendix S3** Free-text comments from healthcare professionals after their scanning sessions

| **Chlorine Dioxide** | **UV-C** |
| --- | --- |
| Not very eco-friendly - so many pairs of gloves and time consuming | The fact that the process is automated removes operator error with Covid |
| Very time consuming | It is the way forward |
| Tristel trio time consuming, not environmentally friendly. | This technique is superior in every respect. time and motion and reproducibility. |
| Tristel - not environmentally friendly and far more subjective | Easy to use machine, better for environment |
| Tristel very inefficient | Love it! |
| Germitec far more efficient; can do other work in the interim | Excellent ease of use |
| So many pairs of gloves! | Very efficient |
| Worry about chemicals and adverse effect on the environment | More environmentally friendly and efficient |
| Not very eco-friendly - time consuming | Much faster, more economy of movement, more automated than Tristel |
| Trusted takes too long and is too involved | Time efficient |
| Not very eco-friendly. Time consuming | We love Germitec |
| Difficult fiddley system with poor traceability | It’s amazing |
| Tristel is time consuming and not environmentally friendly. It’s only as good as the person who does the cleaning regimen. Cleanliness is not guaranteed | We love this product |
| Time inefficient | Fantastic machine, minimal fuss, |
| Time consuming and not very eco | Really easy to use |
| Too time consuming and labour intensive and damages clothes | Very easy to use! Prefer this so much to the wipes |
| Time consuming and poor record keeping | Ease of use, simplicity, environmentally sound |
| Tristel is only really time efficient if you have an assistant to clean the probe which in itself is not efficient |  |
| Not eco-friendly. |  |
| Time consuming. |  |
| Germitec much easier to use. Makes a huge difference to ability to do other things in the clinic |  |
